# Appropriateness of colonoscopy requests according to EPAGE-II in the Spanish region of Catalonia

**DOI:** 10.1186/s12875-015-0369-8

**Published:** 2015-10-26

**Authors:** M. Marzo-Castillejo, J. Almeda, JJ Mascort, O. Cunillera, R. Saladich, R. Nieto, P. Piñeiro, M. Llagostera, FX. Cantero, M. Segarra, D. Puente

**Affiliations:** Unitat de Suport a la Recerca – IDIAP Jordi Gol Direcció d’Atenció Primària Costa de Ponent, Catalan Institute of Health (ICS), c/ Bellaterra, 41 1ª planta, 08940 Cornellà de Llobregat Barcelona, Spain; Unitat de Suport a la Recerca – IDIAP Jordi Gol Direcció d’Atenció Primària Costa de Ponent. Catalan Institute of Health (ICS), CIBER Epidemiología y Salud Pública (CIBERESP), Barcelona, Spain; EAP Florida, UGEAP L’Hospitalet Nord, Universitat de Barcelona, Parc dels ocellets s/n L’Hospitalet de Llobregat, 08905 Barcelona, Spain; EAP Vinyets Ronda Sant Ramon, 187 Sant Boi de Llobregat, 08830 Barcelona, Spain; EAP Penedès Rural. Servei Atenció Primària Alt Penedès.Garraf- Baix Llobregat Nord, Catalan Institute of Health (ICS) Pg. Fluvial s/n, 08730 Sta Margarida i els Monjos Barcelona, Spain; EAP Penedès Rural Servei d’Atenció Primària (SAP) Penedès-Garraf-Llobregat, Nord Catalan Institute of Health (ICS), Plaça Penedès 3, 1ª planta, 08720 Vilafranca del Penedès Barcelona, Spain; EAP Lluis Millet, C/ Lluis Millet, 2-4, 08950 Esplugues de Llobregat Barcelona, Spain; EAP Igualada Urbà. Servei Atenció Primària Anoia. Gerència Territorial Catalunya Central, Catalan Institute of Health (ICS), Pg. Verdaguer, 170, 08700 Igualada Barcelona, Spain; EAP Bellvitge, C. de l’Ermita de Bellvitge, s/n, 08907 L’Hospitalet de Llobregat Barcelona, Spain; Institut Universitari D’Investigació en Atenció Primària Jordi Gol (IDIAP Jordi Gol), Gran Via de les Corts Catalanes, 587 àtic, 08007 Barcelona, Spain

**Keywords:** Colonoscopy, Standards, Colonic diseases

## Abstract

**Background:**

In a context of increasing demand and pressure on the public health expenditure, appropriateness of colonoscopy indications is a topic of discussion. The objective of this study is to evaluate the appropriateness of colonoscopy requests performed in a primary care (PC) setting in Catalonia.

**Methods:**

Cross-sectional descriptive study. Out-patients >14 years of age, referred by their reference physicians from PC or hospital care settings to the endoscopy units in their reference hospitals, to undergo a colonoscopy. Evaluation of the appropriateness of 1440 colonoscopy requests issued from January to July 2011, according to the EPAGE-II guidelines (European Panel on the Appropriateness of Gastrointestinal Endoscopy).

**Results:**

The most frequent indications of diagnostic suspicion requests were: rectal bleeding (37.46 %), abdominal pain (26.54 %), and anaemia study (16.78 %). The most frequent indications of disease follow-up were adenomas (58.1 %), and CRC (31.16 %). Colonoscopy was appropriate in 73.68 % of the cases, uncertain in 16.57 %, and inappropriate in 9.74 %. In multivariate analysis, performed colonoscopies reached an OR of 9.9 (CI 95 % 1.16–84.08) for qualifying as appropriate for colorectal cancer (CRC) diagnosis, 1.49 (CI 95 % 1.1–2.02) when requested by a general practitioner, and 1.09 (CI 95 % 1.07–1.1) when performed on women.

**Conclusions:**

Appropriateness of colonoscopy requests in our setting shows a suitable situation in accordance with recognized standards. General practitioners contribute positively to this appropriateness level. It is necessary to provide physicians with simple and updated guidelines, which stress recommendations for avoiding colonoscopy requests in the most prevalent conditions in PC.

## Background

Colonoscopy is an endoscopic examination which enables accurate location of lesions, and obtaining biopsies. Most conditions that affect the lower gastrointestinal tract can be diagnosed by colonoscopy, and some therapeutic procedures may be performed simultaneously [1]. Colonoscopy is the most sensitive and specific test to detect adenomas and colorectal cancer (CRC).

Colonoscopy requests have significantly increased over the last years as a consequence to the efficacy shown in the CRC screening. [2, 3]. Colonoscopy is considered the test of choice in high risk patients (family history of CRC and/or personal history of advanced adenomas, and is the diagnostic confirmation test when the faecal occult blood test (FOBT) is positive [4].

In developed countries, CRC represents the second cause of incidence and mortality for cancer, both in men and women, occupying the first place when considering both sexes [5]. In Spain, in 2012, CRC was diagnosed in a total of 32,240 cases (19,261 men, and 12,979 women), and was responsible for the death of 14,700 people (8,742 men and 5,958 women) [5]. EUROCARE-5 study results for Spain, estimate a 5-year survival rate for CRC of 57.1 (CI 95 % 56.1–58.1) and a 5-year survival rate for rectal cancer of 56.4 (CI 95 % 55.0–57.7) [6].

Long term results from the *National Polyp Study* confirmed that adenoma removal in high risk CRC patients reduces incidence and mortality for CRC [7, 8]. In contrast, follow-up strategies are not clearly established in patients with low-risk adenomas. Moreover, published studies show that some patients with high-risk CRC do not receive adequate follow-up, compared to a great deal of low-risk CRC patients, who undergo too many colonoscopies [9, 10].

Colonoscopy is indicated to evaluate signs and symptoms of CRC suspicion (rectal bleeding, changes in bowel habit, abdominal pain, anaemia, etc.), which are common reasons for primary care (PC) consultation, and are usually caused by benign limited conditions [11]. Adequate management of patients presenting these symptoms, should involve an initial assessment which considers the balance between necessity of knowing the symptoms aetiology and the performance of a colonoscopy, taking into consideration age and accompanying symptoms.

Colonoscopy, either used as a screening test, a diagnosis confirmation tool, or a therapeutic procedure, is not free from complications [12, 13]. Its diagnostic cost-effectiveness improves with a correct bowel preparation, and when performed under sedation [12, 13]. Colonoscopy cost-effectiveness is also correlated to the health facilities and availability of trained staff. These factors affect the quality offered by the different endoscopy units which, therefore, is not always homogeneous [12, 13].

Open-access endoscopy units allow physicians to request colonoscopies directly, without previous consultation to the gastroenterologist. In a context of increasing demand and pressure on the public health expenditure, appropriateness of colonoscopy indications is a topic of discussion. Various scientific institutions as the *European Panel of Appropriateness of Gastrointestinal Endoscopy* (EPAGE) [14] and *the American Society for Gastrointestinal Endoscopy (ASGE)* [15], have developed different guidelines on the appropriate indication of colonoscopy. The general practitioner, for his/her closeness and accessibility to the public, for his/her continuity of assistance, and for the characteristics of the health problems he/she is involved with, plays a key role in the rational use of colonoscopy.

In our PC setting, no studies on evaluation of the appropriateness of colonoscopy have been published to date. It is for this reason that the following study has been presented, its objectives being: 1) To evaluate the appropriateness of colonoscopy requests issued on a PC setting in Catalonia, according to the EPAGE-II appropriateness guidelines, and 2) To evaluate the possible associations of appropriateness of colonoscopy with the indication of colonoscopy, professional who makes the request for the colonoscopy, reason for request and results.

## Methods

### Design

Cross-sectional descriptive study.

### Setting

Two health districts in Catalonia (the South-Metropolitan and Central Catalonia districts) assigned to the following participating reference Hospitals: Hospital Universitari de Bellvitge, Hospital de Viladecans, Hospital Comarcal de l’Alt Penedès, Hospital de Sant Joan Despí, Moisès Broggi, Hospital General de L’Hospitalet and Hospital General d’Igualada.

### Period study

From January 2011 to July 2011.

### Population sample

Out-patients >14 years of age referred by their reference physicians (gastroenterologists, surgeons, and general practitioners), from primary care or hospital settings to the endoscopy units in their reference hospitals, to undergo a colonoscopy. Neither colonoscopy requests issued from the public health CRC screening program, introduced in some areas where the study was implemented, nor requests from hospital in-patients were included.

### Sample calculation

A sample of 1440 colonoscopy requests was required to determine an appropriateness level of at least 50 %, as situation of maximum uncertainty, with an absolute precision of 4 % and a 95 % confidence interval. A design effect of 1.9 was determined on the cluster sampling (hospitals), and a 20 % of requests were considered ineligible. (Data analysis was performed using the epidemiological data analysis program Epidat 3.1; *Dirección Xeral de Innovación e Xestión da Saúde Pública. Conselleria de Sanidade. Xunta de Galicia*).

### Data collection

All colonoscopy requests which met the inclusion criteria were collected systematically, from the available request lists at every participating reference hospital, in chronological order, up to a maximum of 250 requests per hospital. Information regarding patient characteristics and request indication was obtained from the colonoscopy requests. This information was complemented with the electronic patient records, from which colonoscopy results were also obtained. All information was collected by health professionals (physicians and nurses) using an optical data collection sheet (Teleform 4.0 for Windows, Cardiff Software, Inc., Solana Beach, CA, 1996).

### Main outcome

Appropriateness of colonoscopies was decided according to the EPAGE-II criteria. Evaluation was performed by peer review, with the participation of a first reviewer (a clinician), different in every hospital, and a second reviewer (an epidemiologist) common to all hospitals, with a final agreement by consensus, for discrepancy cases. EPAGE criteria were developed and validated by a multidisciplinary panel of 14 European experts, in 1998, and later reviewed in 2009 (EPAGE-II criteria). EPAGE-II criteria classify indications for colonoscopy into 11 sections, which are scored from 1 (extremely inappropriate) to 9 (extremely appropriate) depending on clinical indication, age, personal and family history. These criteria classify appropriateness of colonoscopy into 3 possible categories: appropriate (≥7), uncertain (4–6), and inappropriate (≤3) [14]. Besides the classification according to the EPAGE-II criteria, the variable appropriateness of colonoscopy was transformed into a dichotomous variable, with a cut-off point of 7 (<=6 inappropriate request; > = 7 appropriate request).

### Other studied variables

Variables on the social and demographic patient characteristics, clinician requesting the procedure, clinical indication of request, results report, diagnosis, and diagnosis relevance were collected. Relevant diagnoses, for this study, were established as: CRC, adenomas, diverticular disease, and intestinal inflammatory disease (IID), which includes ulcerative colitis and Crohn’s disease. The whole set of variables can be consulted on the published study protocol [16].

### Statistical analysis

A descriptive analysis was performed on patient characteristics, clinician requesting the procedure, clinical indication of colonoscopy request, and relevance of the identified lesions. Appropriateness level was determined according to the EPAGE-II criteria (appropriate, uncertain or inappropriate request) globally, and for each indication.

A bivariate analysis for appropriateness of colonoscopy was performed on two categories (cut-off point of 7 on the EPAGE-II scoring system), according to patient factors, requesting clinician factors and diagnosis relevance. Differences between groups were assessed using a *χ*2 test or a Fisher’s exact test, depending on the applying conditions, to compare categorical variables, and Student’s t-test or nonparametric tests (Mann–Whitney U test) to compare continuous variables, according to the variable normal distribution. A 5 % level of significance was used in all contrasts.

Subsequently, a multivariate analysis by multilevel logistic regression was conducted, to assess variables associated to appropriateness, in two categories, after stratification for hospital. For that, the independent variables from the bivariate analysis, which represented a p value ≤0.10 or that were of clinical relevance according to the literature, were considered. In addition, a final model was obtained by progressively excluding those variables which did not provide variability, according to the Akaike information criterion.

Finally, a secondary analysis to establish the correlation between the initial score (first reviewer) and final score (consensus) on the peer review was performed by the intraclass correlation coefficient, considering the cut-off point of 0.75 proposed by Fleiss [17]. This analysis was complemented with the Spearman’s rank correlation coefficient, applying the criteria for quantitative significance recommended by Burnand et al.: *r* < 0.30 = insignificant; 0.30–0.45 = moderate; 0.45–0.60 = substantial; and *r* > 0.60 = high [18]. Statististical analysis was conducted using statistical packages implemented in environment R v.2.14.2.

### Ethics issues

The study protocol was approved to the ethics committee of the Clinical Research Ethics Commitee of the University Institute in Primary Care Research Jordi Gol (IDIAP Jordi Gol) (http://www.idiapjgol.org/). Individualized informed consent was not obtained from the participating patients, because it was not considered necessary according to national regulations [19]. Information was collected anonymously from colonoscopies request forms and computerized records by health care professionals in each centre.

## Results

A total of 1478 colonoscopies were included over the 6 months the study took place. As shown in Table 1, each reference hospital provided from 241–250 colonoscopies (H1-H6). Average patient age was 59.6 years and 50.06 % were women. Regarding its origin, 54.66 % of the requests were issued from primary care centres (PCC). The highest percentage of colonoscopies was requested by general practitioners. Percentages on colonoscopy origin of request and type of requesting clinician significantly varied among the different hospitals.

**Table 1:** Characteristics of patients with colonoscopy request, origin of the request, and type of requesting clinician, depending on participating reference hospital. Results are expressed as mean (standard deviation) and n (%)

	Total (*n* = 1478)	H1 (*n* = 245)	H2 (*n* = 245)	H3 (*n* = 250)	H4 (*n* = 250)	H5 (*n* = 247)	H6 (*n* = 241)
Age^a^	59.6 (15.0)	58.9 (15.7)	59.7 (14.4)	59.6 (14.9)	58.7 (14.8)	59.2 (15,1)	61.7 (15,1)
Women	740 (50.06 %)	111 (45.30 %)	122 (49.79 %)	126 (50.40 %)	133 (53.20 %)	124 (50.20 %)	124 (51.45 %)
Origin (gathered)							
SCC and outpatients	662 (44.79 %)	124 (50.61 %)	142 (57.95 %)	137 (54.80 %)	68 (27.20 %)	121 (49,98 %)	70 (29,04 %)
PCC	808 (54.66 %)	121 (49.38 %)	102 (41.63 %)	113 (45.20 %)	179 (71.60 %)	122 (49,39 %)	171 (70.95 %)
Requesting clinician							
General practitioner	790 (53.45 %)	119 (48.57 %)	95 (38.36 %)	113 (45.20 %)	172 (68.80 %)	121 (48,98 %)	170 (70.53 %)
Gastroenterologist	426 (28.82 %)	21 (8.57 %)	134 (54.69 %)	91 (36.40 %)	65 (26.00)	59 (243,88 %)	56 (23.23 %)
Surgeon	152 (10.28 %)	48 (19.59 %)	8 (3.26 %)	40 (16.00 %)	3 (1.20 %)	40 (16,19 %)	13 (5.18 %)
Internist	27(1.82 %)	16 (6.53 %)	0 (0.00 %)	3 (1.20 %)	0 (0,00 %)	7 (2,83 %)	1 (0.41 %)
Other	51 (3.45 %)	28 (11.42 %)	0 (0.00 %)	3 (1.20 %)	1 (0.40 %)	19 (7,69 %)	0 (0.00 %)

^a^Age expressed as mean ± standard deviation

*SCC* Specialized care centre, *PCC* Primary Care Centre

Table 2 shows colonoscopy characteristics. Most colonoscopies were requested as routine requests (69.72 %), percentage which differs significantly depending on the reference hospital. The highest percentage of colonoscopies responds to the indication of diagnostic suspicion (64.47 %), whilst opportunistic screening represents the lowest request percentage (11.50 %). The most frequent indication of diagnostic suspicion request was rectal bleeding (37.46 %), followed by abdominal pain (26.54 %), and anaemia screening (16.78 %). Among follow-up colonoscopies, the most frequent indication was follow-up of adenomas (58.1 %) and follow-up of CRC (31.16 %), although these percentages were heterogeneously distributed at the different participating hospitals. We have found significant differences between GP and gastroenterologists (<0.001) and between GP and surgeons (<0.039). Although the internist’s adequacy have higher percentage being compared to GP, the differences are not significant (*p* = 0.346).

 According to the EPAGE II criteria, colonoscopies were evaluated as appropriate in 73.68 % of the patients (1089/1478; CI 95 % 71.4–75.9), uncertain in 16.57 % (245/1478; CI 95 % 14.7–18.5), and inappropriate in 9.74 % (144/1478; CI 95 % 8.3–11.4). As detailed in Table 3, appropriateness of colonoscopy respectively increased in relation to age (85.85 % in patients >50 years vs. 39.43 % in ≤50 years), priority of request (81.70 % in urgent priority vs. 70.51 % in routine priority), and clinical indication (79.32 % for diagnostic suspicion vs. 60 % for previously diagnosed condition follow-up). Changes in bowel habit and anaemia were the indications for diagnostic suspicion which presented the highest percentages of appropriateness of colonoscopy. Section ‘Other’, which includes constitutional syndrome and abdominal mass, among other, reached 100 % of appropriateness. Follow-up of CRC reached an EPAGE-II ≥7 in 77.27 % of cases, whereas adenomas only in 53.65 %, despite being the most frequent indication for follow-up (57.74 %). Regarding the type of requesting clinician, general practitioners (76.96 %) showed an EPAGE-II ≥7, slightly higher than the rest of professionals. When comparing the results of diagnostic appropriateness of colonoscopy by type of professional for each of the three categories of indication (diagnostic suspicion, screening and follow-up), the internists achieved the highest percentage in the diagnostic suspicion (92 %), followed by GP (80.8 %). The only significant differences occur when comparing the GP with surgeons (70.3 %) (*p* = 0.011).

**Table 2:** Colonoscopy characteristics depending on participating reference hospital. Results are expressed as n (%)

	Total (*n* = 1478)	H1 (*n* = 245)	H2 (*n* = 245)	H3 (*n* = 250)	H4 (*n* = 250)	H5 (*n* = 247)	H6 (*n* = 241)	*P*
Priority^a^								<0,001
Routine	919 (69.72 %)	171 (73.7 %)	195 (89.9 %)	70 (49.0 %)	221 (89.5 %)	129 (53.3 %)	133 (56,1 %)	
Preferential	235 (24.65 %)	47 (20.3 %)	8 (3.69 %)	45 (31.5 %)	17 (6.88 %)	76 (31.4 %)	42 (17,7 %)	
Urgent	164 (17.20 %)	14 (6.03 %)	14 (6.45 %)	28 (19.6 %)	9 (3.64 %)	37 (15.3 %)	62 (26,2 %)	
Indication								0,019
Diagnostic suspicion	953 (64.47 %)	164 (66.9 %)	152 (62.0 %)	156 (62.4 %)	165 (66.0 %)	152 (61.5 %)	164 (68,0 %)	
Follow-up	355 (24.01 %)	59 (24.1 %)	53 (21.6 %)	66 (26.4 %)	47 (18.8 %)	70 (28.3 %)	60 (24,9 %)	
Opportunistic screening	170 (11.50 %)	22 (8.98 %)	40 (16.3 %)	28 (11.2 %)	38 (15.2 %)	25 (10.1 %)	17 (7,05 %)	
Type of diagnostic suspicion^b^								0,012
Rectal bleeding	357 (37.46 %)	53 (32.3 %)	60 (39.5 %)	55 (35.3 %)	71 (43.0 %)	60 (39.5 %)	58 (35,4 %)	
Abdominal pain	253 (26.54 %)	48 (29.3 %)	42 (27.6 %)	37 (23.7 %)	36 (21.8 %)	32 (21.1 %)	58 (35,4 %)	
Anaemia	160 (16.78 %)	30 (18.3 %)	21 (13.8 %)	32 (20.5 %)	27 (16.4 %)	26 (17.1 %)	24 (14,6 %)	
Changes in bowel habit	76 (7.97 %)	20 (12.2 %)	13 (8.55 %)	18 (11.5 %)	13 (7.88 %)	10 (6.58 %)	2 (1,22 %)	
Other	107 (11.22 %)	13 (7.93 %)	16 (10.5 %)	14 (8.97 %)	18 (10.9 %)	24 (15.8 %)	22 (13,4 %)	
Type of follow-up^c^								<0,001
Adenomas	205 (58.07 %)	22 (37.9 %)	34 (64.2 %)	37 (56.9 %)	37 (78.7 %)	32 (45.7 %)	43 (71,7 %)	
CRC	110 (31.16 %)	29 (50.0 %)	14 (26.4 %)	19 (29.2 %)	6 (12.8 %)	35 (50.0 %)	7 (11,7 %)	
Ulcerative colitis	16 (4.53 %)	3 (5.17 %)	3 (5.66 %)	2 (3.08 %)	2 (4.26 %)	2 (2.86 %)	4 (6,67 %)	
Crohn’s disease	7 (1.98 %)	1 (1.72 %)	0 (0.00 %)	5 (7.69 %)	0 (0.00 %)	1 (1.43 %)	0 (0,00 %)	
Diverticular disease	3 (0.85 %)	0 (0.00 %)	1 (1.89 %)	0 (0.00 %)	0 (0.00 %)	0 (0,00 %)	2 (3,33 %)	
Other	12 (3.40 %)	3 (5.17 %)	1 (1.89 %)	2 (3.08 %)	2 (4.26 %)	0 (0,00 %)	4 (6,67 %)	

^a^over the total (missing cases excluded)

^b^Over diagnostic suspicion indication

^c^Over follow-up indication

*CRC* Colorrectal cancer

**Table 3:** Classification of colonoscopy appropriateness according to the EPAGE II criteria. Characteristics of the patients, clinicians, and indication of request. Results expressed as n (%). EPAGE II criteria percentages are shown per row

	Total *N* = 1478 (100 %)	≤3 144 (9.7 %)	4-6 245 (16.6 %)	≥7 1089 (73.7 %)	*p* value
Age					<0.001
≤50 years	388 (26.25 %)	33 (8.50 %)	202 (52.06 %)	153 (39.43 %)	
>50 years	1089 (73.68 %)	111 (10.19 %)	43 (3.94 %)	935 (85.85 %)	
Gender					0.145
Women	740 (50.06 %)	66 (8.91 %)	113 (15.27 %)	561 (75.81 %)	
Men	738 (49.93 %)	78 (10.56 %)	132 (17.88 %)	528 (71.54 %)	
Origin (gathered)					<0.001
SCC and outpatients	662 (45.03 %)	86 (12.99 %)	115 (17.37 %)	461 (69.63 %)	
PCC	808 (54.49 %)	58 (7.17 %)	128 (15.84 %)	622 (76.98 %)	
Requesting clinician					0.001
General practitioner	790 (54.63 %)	57 (7.21 %)	125 (15.82 %)	608 (76.96 %)	
Gastroenterologist	426 (29.46 %)	61 (14.31 %)	72 (16.90 %)	293 (68.77 %)	
Internist	27 (1.86 %)	1 (3.70 %)	2 (7.40 %)	24 (88.88 %)	
Surgeon	152 (10.51 %)	18 (11.84 %)	31 (20.39 %)	103 (67.76 %)	
Other	51 (3.52 %)	4 (7.84 %)	10 (19.60 %)	37 (72.54 %)	
Priority					0.001
Routine	919 (69.72 %)	105 (11.42 %)	166 (18.06 %)	648 (70.51 %)	
Preferential	235 (24.01 %)	14 (5.95 %)	35 (14.89 %)	186 (79.14 %)	
Urgent	164 (11.50 %)	6 (3.65 %)	24 (14.63 %)	134 (81.70 %)	
Indication					<0.001
Diagnostic suspicion	953 (64.47 %)	14 (1.46 %)	186 (19.51 %)	753 (79.32 %)	
Follow-up	355 (24.01 %)	117 (32.95 %)	25 (7.04 %)	213 (60.00 %)	
Opportunistic screening	170 (11.50 %)	13 (7.64 %)	34 (20.00 %)	123 (72.35 %)	
Type of diagnostic suspicion^a^					<0.001
Rectal bleeding	357 (37.46 %)	0 (0.00 %)	106 (29.69 %)	251 (70.30 %)	
Abdominal pain abdominal	253 (26.54 %)	1 (0.39 %)	73 (28.85 %)	179 (70.75 %)	
Anaemia	160 (16.78 %)	9 (5.62 %)	7 (4.37 %)	144 (90.00 %)	
Changes in bowel habit deposicional	76 (7.97 %)	4 (5.26 %)	0 (0.00 %)	72 (94.73 %)	
Other	107 (11.22 %)	0 (0.00 %)	0 (0.00 %)	107 (100 %)	
Type of follow-up^b^					<0.001
Adenomas	205 (57.74 %)	85 (41.46 %)	10 (4.87 %)	110 (53.65 %)	
CRC	110 (30.98 %)	12 (10.90 %)	13 (11.81 %)	85 (77.27 %)	
Ulcerative colitis	16 (4.50 %)	9 (56.25 %)	1 (6.25 %)	6 (37.50 %)	
Crohn’s disease	7 (1.97 %)	1 (14.28 %)	0 (0.00 %)	6 (85.71 %)	
Diverticular disease	3 (0.84 %)	0 (0.00 %)	0 (0.00 %)	3 (100.00 %)	

^a^Over diagnostic suspicion indication

^b^Over follow-up indication

*CRC* Colorectal cancer

Out of the 1.478 colonoscopies, a total of 1.975 diagnoses were established (Table 4). In 388 cases (19.64 %) colonoscopy was normal, in 594 cases (30.07 %) haemorrhoids were diagnosed, and in 51.27 % a relevant diagnosis was confirmed. Distribution of these diagnoses showed significant differences between reference hospitals.

**Table 4:** Colonoscopy results depending on participating reference hospital. Results are expressed as n (%)

		Reference Hospitals						
Type of Diagnosis	Total (*n* = 1975)	H1 (*n* = 310)	H2 (*n* = 300)	H3 (*n* = 412)	H4 (*n* =312)	H5 (*n* = 352)	H6 (*n* = 289)	*P*
Normal	388 (19.64 %)	49 (15.81 %)	68 (22.67 %)	31 (7.52 %)	101 (32.37 %)	48 (13.64 %)	91 (31.49 %)	<0.001
Haemorrhoids	594 (30.07 %)	130 (41.94)	109 (36.33 %)	130 (31.55 %)	45 (14.42 %)	133 (37.78 %)	47 (16.26 %)	<0.001
Adenomas	377 (19.08 %)	49 (15.81 %)	70 (23.33 %)	88 (21.36 %)	56 (17.95 %)	75 (31.31 %)	39 (13.49 %)	<0.001
Diverticular disease	297 (15.03 %)	51 (16.45 %)	32 (10.67 %)	42 (10.19 %)	44 (14.10 %)	67 (19.03 %)	61 (21.11 %)	0.001
CRC	46 (2.32 %)	8 (2.58 %)	10 (3.33 %)	8 (1.94 %)	7 (2.24 %)	6 (1.70 %)	7 (2.42 %)	0.932
IID	24 (1.21 %)	5 (1.61 %)	6 (2.00 %)	6 (1.46 %)	0 (0.00 %)	4 (1.14 %)	3 (1.04 %)	0.129
Other diagnosis	249 (12.60 %)	18 (5.81 %)	5 (1.67 %)	107 (25.97 %)	59 (18.91 %)	19 (5.40 %)	41 (14.19 %)	<0.001

*CRC* Colorectal cancer, *IID* Inflammatory Intestinal disease (includes ulcerative colitis. and Crohn’s disease)

Table 5 presents the EPAGE-II criteria distribution, according to the reached diagnoses. 97.82 % of colonoscopies with a diagnosis of CRC obtained an adequate EPAGE-II score. This value stands close to 55 % in the IID. In addition, 70.36 % of colonoscopies with normal results or with irrelevant diagnosis showed an adequate EPAGE-II.

**Table 5:** Distribution of EPAGE II criteria depending on colonoscopy results. Results are expressed as n (%). EPAGE II criteria percentages are shown per row

	Total (*n* = 1478)	≤3 (*n* = 144)	4-6 (*n* = 245)	≥7 (*n* = 1089)	*p* value
	*N* = 1975	197	301	1477	
Normal	388 (19.6426.31 %)	40 (10.30 %)	75 (19.32 %)	273 (70.36 %)	0.134
Haemorrhoids	594 (30.07 %)	44 (7.40 %)	114 (19.19 %)	436 (73.40 %)	0.008
Adenomas	377 (19.08 %)	51 (13.52 %)	32 (8.48 %)	294 (77.98 %)	<0.001
Diverticular disease	297 (15.03 %)	36 12.12 %)	27 (9.09 %)	234 (78.78 %)	<0.001
CRC	46 (2.32 %)	0 (0.00 %)	1 (2.17 %)	45 (97.82 %)	<0.001
IID	24 (1.21 %)	5 (20.83 %)	6 (25.00 %)	13 (54.16 %)	0.056
Other	249 (12.60 %)	21 (8.43 %)	46 (18.44 %)	182 (73.09 %)	0.387

*CRC* Colorectal cancer, *IID* Inflammatory Intestinal disease (includes ulcerative colitis, and Crohn’s disease)

*p*-value corresponds to the Fisher’s exact test

All included variables shown significant differences on the bivariate analysis of appropriateness of colonoscopies (two categories, cut-off point ≥7), except for gender, normal colonoscopy diagnosis, haemorrhoids, and ‘other diagnoses’. In the multivariate and multilevel analysis final model (Table 6), performed colonoscopies reached an odds ratio (OR) of qualifying as appropriate for CRC diagnosis of 9.9 (OR = 9.87; CI 95 % 1.16–84.08), 1.5 (OR = 1.49; CI 95 % 1.1–2.02) if the colonoscopy request was issued by a general practitioner, and 1.1 (OR = 1.09; CI 95 % 1.07–1.1) if the colonoscopy was performed on women. OR for colonoscopies to be classified as appropriate, increased nearly 10 % for every one year increase in patient age (OR 1.09; CI 95 % 1.07–1.10). On the other hand, OR for colonoscopies to be classified as appropriate, decreased if the indication for request was follow-up (OR 0.23; CI 95 % 0.14–0.38). Colonoscopy request for diagnostic suspicion, and presence of adenomas or IID as colonoscopy result, were explicative variables (according to the Akaike Information Criteria) in the model but not statistically significant.

**Table 6:** Multilevel logistic regression models of appropriateness of colonoscopy, assuming individuals as first level, and hospitals as second level. Results are expressed as OR (CI 95 %)

	Complete Model	AIC Model
Independent variable	0.02 (0.01, 0.04)	0.02 (0.01, 0.04)
Age	1.09 (1.07, 1.1)	1.09 (1.07, 1.1)
Gender		
Men	Ref.	Ref.
Women	1.09 (1.07, 1.1)	1.09 (1.07, 1.1)
Type of requesting clinician		
Other	Ref.	Ref.
General practitioner	1.49 (1.1, 2.02)	1.49 (1.1, 2.02)
Indication		
Screening	Ref.	Ref.
Diagnostic suspicion	1.21 (0.77, 1.89)	1.2 (0.77, 1.88)
Follow-up	0.23 (0.14, 0.38)	0.23 (0.14, 0.38)
Priority		
Routine	Ref.	Ref.
Preferential	1.37 (0.89, 2.1)	1.35 (0.88, 2.07)
Urgent	1.57 (0.92, 2.67)	1.57 (0.92, 2.67)
Colonoscopy result		
Adenoma	1.39 (0.97, 2)	1.4 (0.97, 2.01)
CRC	9.87 (1.16, 84.08)	10.07 (1.19, 85.33)
IID	2.54 (0.86, 7.51)	2.54 (0.86, 7.49)
Diverticular disease	0.92 (0.62, 1.35)	-
Haemorrhoids	0.96 (0.7, 1.31)	-
Other	1.03 (0.68, 1.57)	-

*CRC* Colorectal cancer, *IID* Inflammatory Intestinal disease (includes ulcerative colitis, and Crohn’s disease), *Ref* reference, *AIC Model* Akaike’s Information Criterion

Despite the variable origin of the colonoscopy request showed significant differences on the bivariate analysis, this variable was not included in the logistic regression model due to the interaction and multicollinearity with the colonoscopy requesting clinician. Concordance between the EPAGE-II score assessed by the first reviewer and the final score obtained by consensus, presented an intraclass correlation coefficient of 0.704 (moderate-good), and a Spearman’s rank correlation coefficient of 0.651.

## Discussion

The study results show that, following the EPAGE-II guidelines, the indications of colonoscopy are appropriate in a high percentage (73.68 %), although, the percentage of uncertain, and inappropriate indications (16.57 % and 9.74 % respectively), are not negligible. These results are comparable to those obtained in the individual studies considered in the systematic review by Hassan et al. [20], where the estimated percentage of uncertain and inappropriate colonoscopies was 26 %. This review included 12 cohort studies (14.160 colonoscopies) performed in countries of Europe and Asia, and assessed the appropriateness of colonoscopy according to the EPAGE-II and ASGE criteria [20].

Results on inappropriateness of colonoscopy vary among the various countries and published studies. A multicentric study with the participation of 11 European countries (5,213 colonoscopies), using EPAGE criteria, positions inappropriateness of colonoscopy in 27 % [21]. Studies conducted in Switzerland situate it at 13–27 % [22–25], and studies performed in Spain, at 23–31 % [26, 27]. Studies published from 2009 onwards, used the EPAGE-II criteria, and obtained far more satisfactory results. Therefore, the studies conducted in Spain, based on the EPAGE-II, situate inappropriateness at 10.5–17.5 % [28–30], closer to the inappropriateness results of our study (9.7 %).

The percentage of uncertain colonoscopies in our study (16.57 %) stands in an average place among the studies conducted in our setting, which used the EPAGE-II criteria (between 9.1 % and 18 %) [28–30]. Studies conducted using the EPAGE criteria exhibit a considerably higher percentage of uncertain colonoscopies. In fact, the wide number of situations considered as uncertain by the EPAGE criteria, was the reason for applying the new EPAGE-II criteria in our study [14].

In our study, colonoscopies requested by general practitioners exhibited good appropriateness. These results are similar to those published in some studies in our setting [27, 30], but are not confirmed in other series where gastroenterologists present better percentages of colonoscopy appropriateness [26]. In any case, our results are not consistent with the statement that inappropriate colonoscopies, according to studies conducted in USA, Europe and the Middle East, increase in endoscopy units that accept patients from PC [31]. However, these studies may not be comparable, due to the differences in the various country health systems, and in the use of protocols and guidelines in PC.

In this regard, the differences in appropriateness of colonoscopy, found in the various hospitals participating in the study, may be explained by type of hospital (tertiary referral hospital -H2, H4, H6- or district hospital -H1, H3, H5-), organization of the various endoscopic units, request priority, waiting lists, delay in the procedure, protocols and circuits, as well as clinician criteria, including PC physicians requesting the colonoscopy. Nevertheless, we do not hold the data that confirm to which extent, these factors may have influenced the appropriateness of colonoscopy results in each endoscopy unit participating in the study.

As expected, colonoscopy diagnostic performance was significantly higher in requests of patients over 50, in urgent requests, and in cases where the indication for request was diagnostic suspicion. In our series, inappropriate indication was especially relevant in follow-up of adenomas, and in follow-up of IID. Colonoscopy uncertain indication, lay with the request for CRC suspicion, namely, rectal bleeding and abdominal pain. These results are comparable to those from studies that used EPAGE-II criteria [28–30].

An adequate colonoscopy indication may contribute to improve prognosis of patients with CRC. However, an adequate appropriateness of colonoscopy does not guarantee a high association with CRC diagnosis. In this regard, our results are similar to those found in other studies [26–30].

Regarding the strengths of this study, we would like to stress the number of colonoscopies included, along with the number of participating endoscopy units, in comparison with other studies conducted in our setting [26–29]. Moreover, we also emphasize the fact that this study evaluation was conducted by peer review, analysing discrepancies and seeking agreement, despite this method entailed, in some cases, double-checking the obtained information from the colonoscopy requests, and from the electronic medical records. Unfortunately, the number of different specialist has not been enough for comparing some of variables categories. For example, the internists’ number who participated in the study was not enough to reach statistical significance when comparing the categories of indication.

Despite results on concordance between the EPAGE-II scores assessed by the first reviewer and final scores obtained by consensus, showed an acceptable index according to generic cut-off points, in the context of correlation between reviewers, they were low values, presenting a large number of discrepancies. We consider these discrepancies may significantly diminish, if all the necessary information to assess the EPAGE-II were available to the reviewers, leaving no scope for interpretation. This limitation of our study is common to the various published studies, and may be avoided if studies were conducted prospectively. Assessing colonoscopy indications prospectively, and concluding whether or not colonoscopy results modified patient management, would provide further elements to determine whether colonoscopy requests were appropriate. However, studies that assess appropriateness of colonoscopy prospectively, obtain similar results [26, 30, 32].

In general, studies on appropriateness of colonoscopy show there is scope for improvement in the indications of colonoscopy. Our study demonstrates need for improvement, especially, in the indications for follow-up of adenomas, and IID, which are relevant and prevalent conditions in the PC setting. Otherwise, we have also confirmed that the EPAGE is not a simple and user friendly tool to be used in the PC practice. In our PC setting, we consider it is essential to provide clinicians with clinical practice guidelines (CPG) and agreed by consensus protocols between the different health care levels, with simple, updated, and easy to implement recommendations, to help adequate decision-making, both in the sense of recommending colonoscopy, as well as not recommending it. Recommendations which will help to control colonoscopy demand, reduce waiting lists, diminish costs, and simultaneously, detect all pathology. In this regard, continuous training of the involved professionals, along with regular sessions where comments and feed-back from the colonoscopy results are discussed, should become crucial objectives. Moreover, appropriateness of colonoscopy requires the availability of quality endoscopy units, along with well- established circuits between PC and specialized care.

In our PC setting, we reckon that the results obtained in our study, especially on the section ‘signs and symptoms of suspicion’ (rectal bleeding, changes in bowel habit, and abdominal pain), respond, partly, to the implementation of the available CPG on the management of rectal bleeding [11], and on prevention of CRC [4]. These CPG were jointly developed by the Spanish Association of Gastroenterology (AEG, by its acronym in Spanish), the Spanish Society for Family and Community Medicine (semFYC, by its acronym in Spanish), and the Iberoamerican Cochrane Centre, which have been widely accepted among clinicians. However, it is necessary to emphasize the need for improvement in the follow-up of adenomas, and comply with the updated protocols on diagnostic and therapeutic management, like those recently proposed by the European Society of Gastrointestinal Endoscopy (ESGE) [33].

## Conclusions

Appropriateness of colonoscopy requests in our setting, assessed by the EPAGE-II, shows a favourable situation, according to the National and International standards. General practitioners contribute positively to this appropriateness level. In order to avoid all those cases of inappropriate colonoscopies, and obtain a higher diagnostic performance, along with reduced costs, and waiting lists, it is necessary to provide physicians with simple and updated guidelines, which also emphasize recommendations on not always requesting colonoscopies in the most prevalent conditions in PC.

## Abbreviations

AEG: Spanish Association of Gastroenterology
ASGE: American Society for Gastrointestinal Endoscopy
CCR: Colorectal cancer
EPAGE: European Panel on the Appropriateness of Gastrointestinal Endoscopy
ESGE: European Society of Gastrointestinal Endoscopy
FOBT: Faecal occult blood test
CPG: Clinical practice guidelines
IID: Intestinal inflammatory disease
PC: Primary care
PCC: Primary care centres
semFYC: Spanish Society for Family and Community Medicine
